# Bovine Trichomonosis Cases in the United States 2015–2019

**DOI:** 10.3389/fvets.2021.692199

**Published:** 2021-08-09

**Authors:** Katy A. Martin, Juli Henderson, Matthew T. Brewer

**Affiliations:** Department of Veterinary Pathology, Iowa State University College of Veterinary Medicine, Ames, IA, United States

**Keywords:** bovine trichomonosis, bovine trichomoniasis, sexually transmitted diseases, flagellate, mucosoflagellate, Parabasalia

## Abstract

*Tritrichomonas foetus* is a sexually-transmitted protozoan parasite that causes early embryonic death in cattle. *Tritrichomonas foetus* is enzootic in the United States but is not a reportable disease at the national level. Thus, it is difficult to understand the prevalence and relative distribution of the disease for the purpose of developing appropriate control measures. In this study, a survey of state veterinarians was used to determine the number of reported cases in each state from 2015 to 2019. Our investigation revealed infections in 25 different states and a total of 3,817 reported cases nationwide. Infections occurred throughout different regions of the country, and numbers of cases were only weakly correlated with total number of cattle in each state. *Tritrichomonas foetus* is a significant pathogen in the United States and understanding the relative distribution of the parasite is useful for prioritizing surveillance and intervention strategies going forward.

## Introduction

Bovine trichomonosis is caused by *Tritrichomonas foetus*, an obligate parasite of the bovine reproductive tract. Pear-shaped trophozoites are transmitted among bulls and cows during coitus. Infected bulls are typically without clinical signs while cows experience metritis and early embryonic death (1, 2). There are currently no drugs approved for the treatment of *T. foetus* in the United States (3, 4). Although most cows ultimately clear the infection, lost pregnancies lead to a prolonged calving period and an increase in open cows (5). This leads to a number of economic consequences for cow-calf producers including reduced uniformity in calf weights and the need to cull a larger number of cows prior to completion of their productive lifespan (6).

Compared to other infectious diseases of veterinary interest, *T. foetus* is expected to have a relatively low prevalence in the United States. Accordingly, control of bovine trichomonosis is not coordinated at the federal level, leaving a patchwork of regulations among states. Each state determines cattle entry requirements including the type of diagnostic assay performed. In order to formulate control strategies that are tailored to needs of different regions, additional data regarding the occurrence of bovine trichomonosis is needed.

Each state employs a state veterinarian whose office is involved with management of diseases in agricultural animals within the state. Thus, the state veterinarian is often a unique resource with information not collected at the federal level. In this study, our goal was to determine the approximate number of known cases of bovine trichomonosis reported in the United States during the past 5 years by surveying state veterinarians.

## Methods

During the summer of 2020, state veterinarians from all 50 US states were contacted to complete a survey regarding the status of *T. foetus in* their state of residence. Institutional review board (IRB) approval was waived by university policy since no individual patients were identified and the information is purely factual. In most cases, the state veterinarian could determine the number of cases detected during the past 5 years. In four cases (California, Florida, Kentucky, and Michigan), we were referred to diagnostic laboratory personnel for case numbers and information. Since *T. foetus* is not reportable in all states, some states were unable to provide any information regarding disease status. State veterinarians were asked to report the number of positive tests, the diagnostic method—culture or PCR,—reporting status, and regulations for *T. foetus* for the years 2015–2019. Total cattle inventories for each state were determined using https://usda-reports.penguinlabs.net. A simple linear regression was used to assess the correlation between total cattle inventory and number of reported cases.

## Results

### At Least 25 Federal States Detected Bovine Trichomonosis in the Past 5 Years

**Figure 1:**
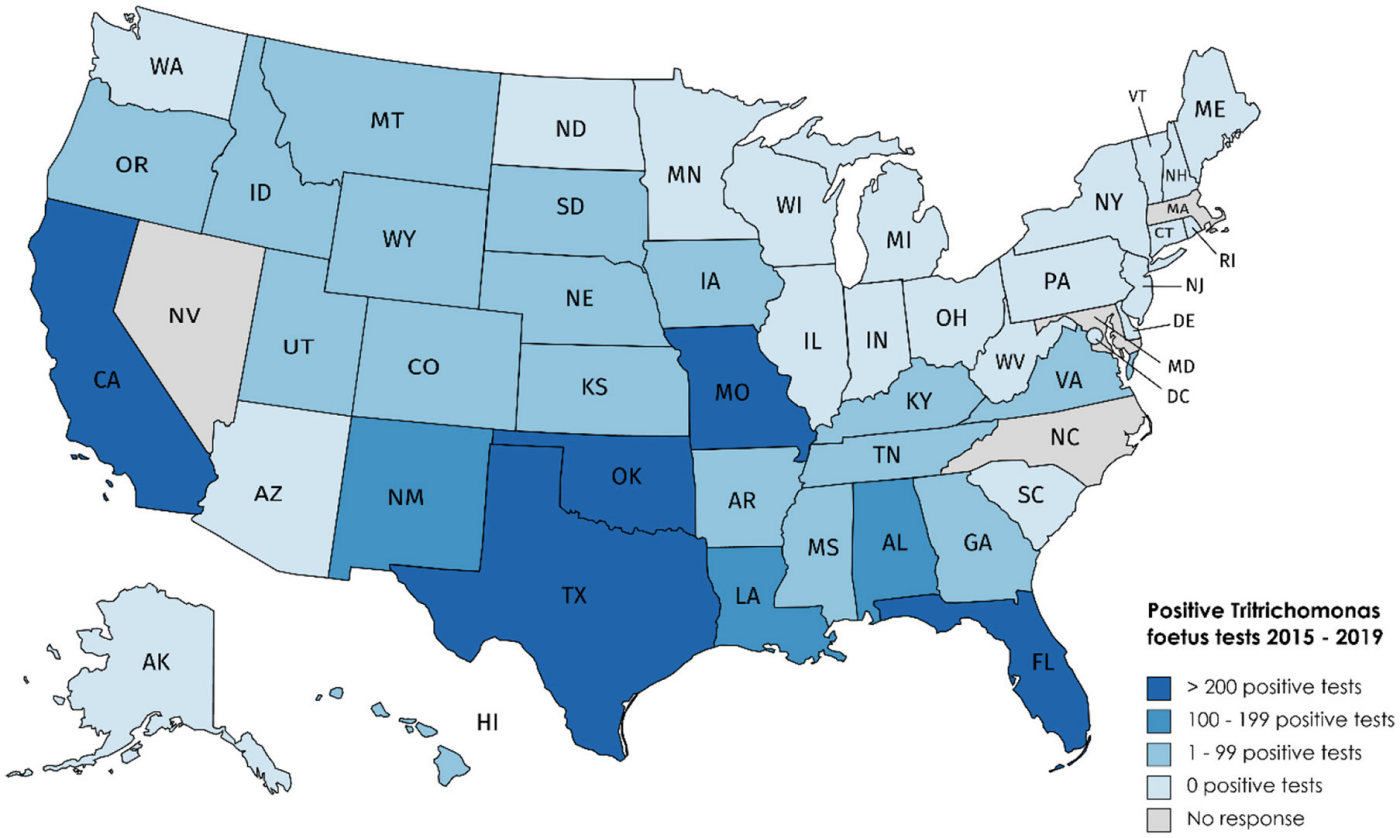
Bovine trichomonosis cases reported 2015–2019.

**Figure 2:**
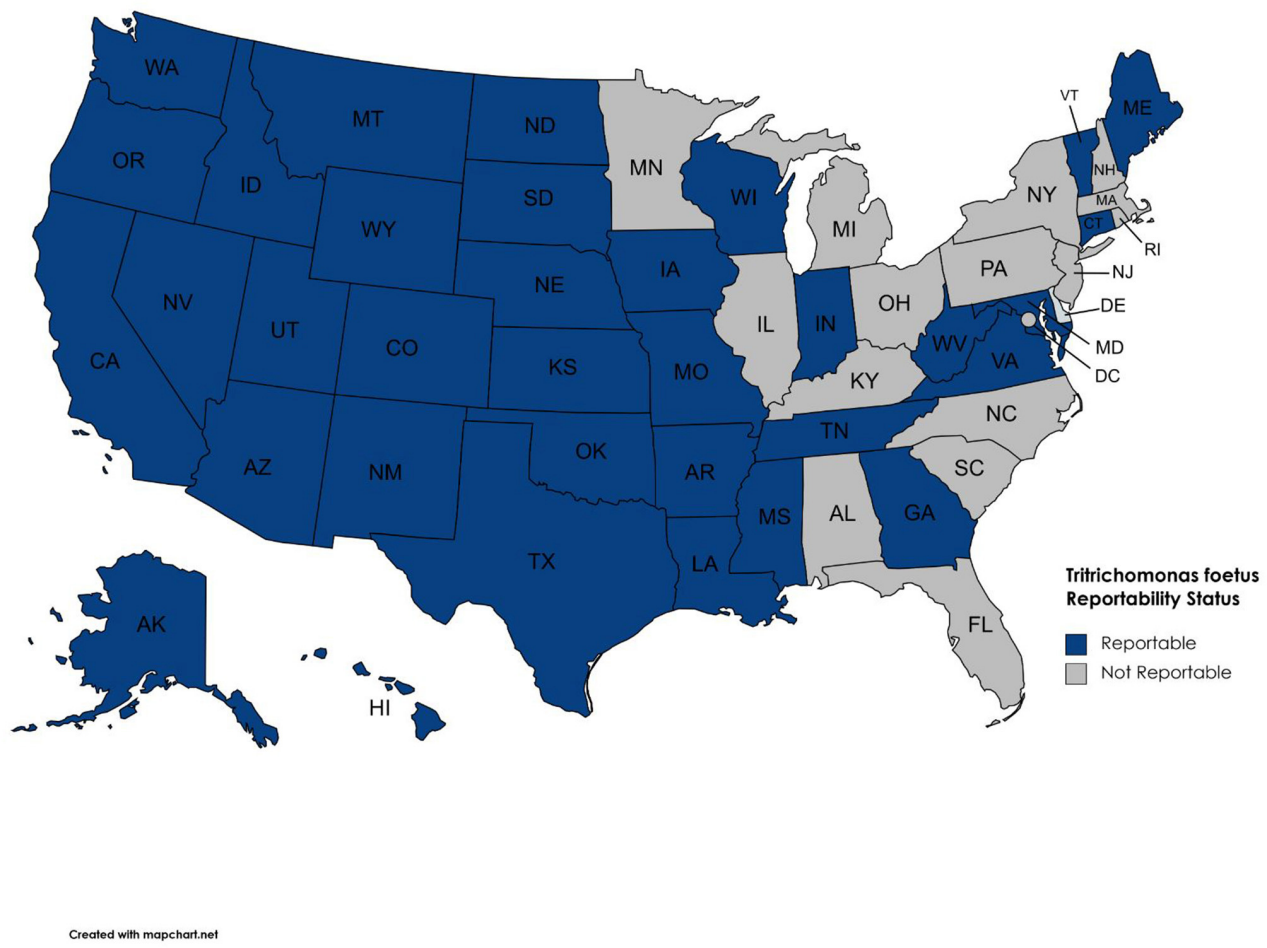
Reportable disease status for *T. foetus* by state.

Our survey revealed 3,817 positive tests reported from 2015 to 2019 (Table 1, Figure 1). There were 25 states with at least one positive case within the last 5 years, while 20 states reported zero cases in the same timeframe. Of the states reporting zero cases, reporting requirements were in place for 10 out of the 20 states. We were unable to obtain case information from 5 states (Arizona, Maryland, Massachusetts, Nevada, and North Carolina); of these states, *T. foetus* was only reportable in Maryland at the time of the survey; it is now reportable in Arizona. Seventeen states do not require reporting of *T. foetus* cases (Figure 2); our survey revealed 446 (11.6%) cases from these states. Texas reported the highest total number of positive tests between 2015 and 2019 (1,468, 38.4%). Of the states reporting positive tests, only 7 reported >100 positives from 2015 to 2019: Texas, Oklahoma (428, 11.2%), California (380, 9.9%), Missouri (283, 7.4%), Florida (262, 6.8%), Louisiana (187, 4.9%), and Alabama (179, 4.7%).

**Table 1:** Cases of bovine trichomonosis reported by state, 2015–2019.

	**2015 (PCR/Culture)**	**2016 (PCR/Culture)**	**2017 (PCR/Culture)**	**2018 (PCR/Culture)**	**2019 (PCR/Culture)**	**Total (PCR/Culture)**	**Reporting requirement?**
Alabama	12 (12/0)	53 (53/0)	32 (32/0)	2 (2/0)	79 (79/0)	179 (179/0)	No
Alaska	0	0	0	0	0	0	Yes
Arizona	ND^*^						No^**^
Arkansas				2 (2/0)	12 (12/0)	14	Yes
California	60 (52/8) (includes 9 positive cows)	65 (53/12)	76 (76/0)	90 (78/12) (includes 12 positive cows)	89 (74/15) (includes 4 positive cows)	380 (333/47)	Yes
Colorado	9 (9/0)	3 (3/0)	2 (2/0)	4 (4/0)	8 (8/0)	26 (26/0)	Yes
Connecticut	0	0	0	0	0	0	Yes
Delaware	0	0	0	0	0	0	Yes
Florida	11 (0/11)	25 (22/3)	52 (52/0)	124 (124/0)	50 (50/0)	262	No
Georgia						20 (19/1)	Yes
Hawaii	4 (4/0)	8 (8/0)	NOT AVAILABLE	5 (5/0)	3 (3/0)	20 (20/0)	Yes
Idaho	3 (3/0)	0	11 (11/0)	2 (2/0)	1 (1/0)	17 (17/0)	Yes
Illinois	0	0	0	0	0	0	No
Indiana	0	0	0	0	0	0	Yes
Iowa	9 (9/0)	6 (6/0)	5 (5/0)	8 (8/0)	5 (5/0)	33 (33/0)	Yes
Kansas	8 (8/0)	3 (3/0)	16 (16/0)	38 (38/0)	14 (14/0)	79 (79/0)	Yes
Kentucky	0	0	4 (4/0) (includes 2 positive cows)	0	1 (1/0)	5 (5/0)	No
Louisiana	15 (15/0)	33 (33/0)	25 (25/0)	30 (30/0)	84 (84/0)	187 (187/0)	Yes
Maine	0	0	0	0	0	0	Yes
Maryland	ND^*^						Yes
Massachusetts	ND^*^						No
Michigan	0	0	0	0	0	0	No
Minnesota	0	0	0	0	0	0	No
Mississippi		24 (24/0)	44 (44/0)	5 (5/0)	22 (22/0)	95 (95/0)	Yes
Missouri	84 (84/0)	96 (96/0)	38	23	42	283 (283/0)	Yes
Montana		6 (6/0)	1 (1/0)	1 (1/0)	1 (1/0)	9 (9/0)	Yes
Nebraska	16 (16/0)	23 (23/0)	2 (2/0)	17 (17/0)	0	58 (58/0)	Yes
Nevada	ND^*^						No
New Hampshire	0	0	0	0	0	0	No
New Jersey	0	0	0	0	0	0	No
New Mexico		84 (84/0)			29 (29/0)	113 (113/0)	Yes
New York	0	0	0	0	0	0	No
North Carolina	ND^*^						No
North Dakota	0	0	0	0	0	0	Yes
Ohio	0	0	0	0	0	0	No
Oklahoma	126 (126/0)	121 (121/0)	76 (76/0)	49 (49/0)	56 (56/0)	428 (428/0)	Yes
Oregon	6 (6/0)	3 (3/0)	2 (2/0)	2 (2/0)	3 (3/0)	16 (16/0)	Yes
Pennsylvania	0	0	0	0	0	0	No
Rhode Island	0	0	0	0	0	0	No
South Carolina	0	0	0	0	0	0	No
South Dakota	2 (2/0)	38 (38/0)	0	2 (2/0)	2 (2/0)	44 (44/0)	Yes
Tennessee	2 (2/0)	0	0	4 (4/0)	4 (4/0)	10 (10/0)	Yes
Texas	446 (439/7)	408 (404/4)	327 (325/2)	285 (285/0)	329 (329/0)	1,468 (1,455/13)	Yes
Utah	7 (7/0)	11 (11/0)	5 (5/0)	0	30 (30/0)	53 (53/0)	Yes
Vermont	0	0	0	0	0	0	Yes
Virginia	0	0	0	0	1 (1/1)^***^	1 (1/1)^***^	Yes
Washington	0	0	0	0	0	0	Yes
West Virginia	0	0	0	0	0	0	Yes
Wisconsin	0	0	0	0	0	0	Yes
Wyoming	14 (0/14)	3 (3/0)	0	0	0	17 (3/14)	Yes

* *ND, Not determined. Cases not reported or no response to survey*.

** *Reporting requirement in place after the time of survey*.

*** *Single case diagnosed by both culture and PCR testing methods*.

### PCR-Based Testing of Bulls Accounts for the Majority of Reported Cases

Table 1 provides the number of cases per year from 2015 through 2019 along with the diagnostic method used (PCR vs. culture). The majority of positive tests were PCR-based; only six states (California, Florida, Georgia, Texas, Virginia, and Wyoming) reported positive cultures. These positive culture tests accounted for 76 (2%) out of 3,817 total positive tests, with the remaining 3,741 (98%) positive tests being PCR results. Only California and Kentucky case data reported infections in cows; all other positive tests originated from bull samples only. Positive cow samples accounted for only 27 (0.71%) out of 3,817 positive cases included in our data.

### Correlation of Cattle Inventory With Trichomonosis Cases

**Figure 3:**
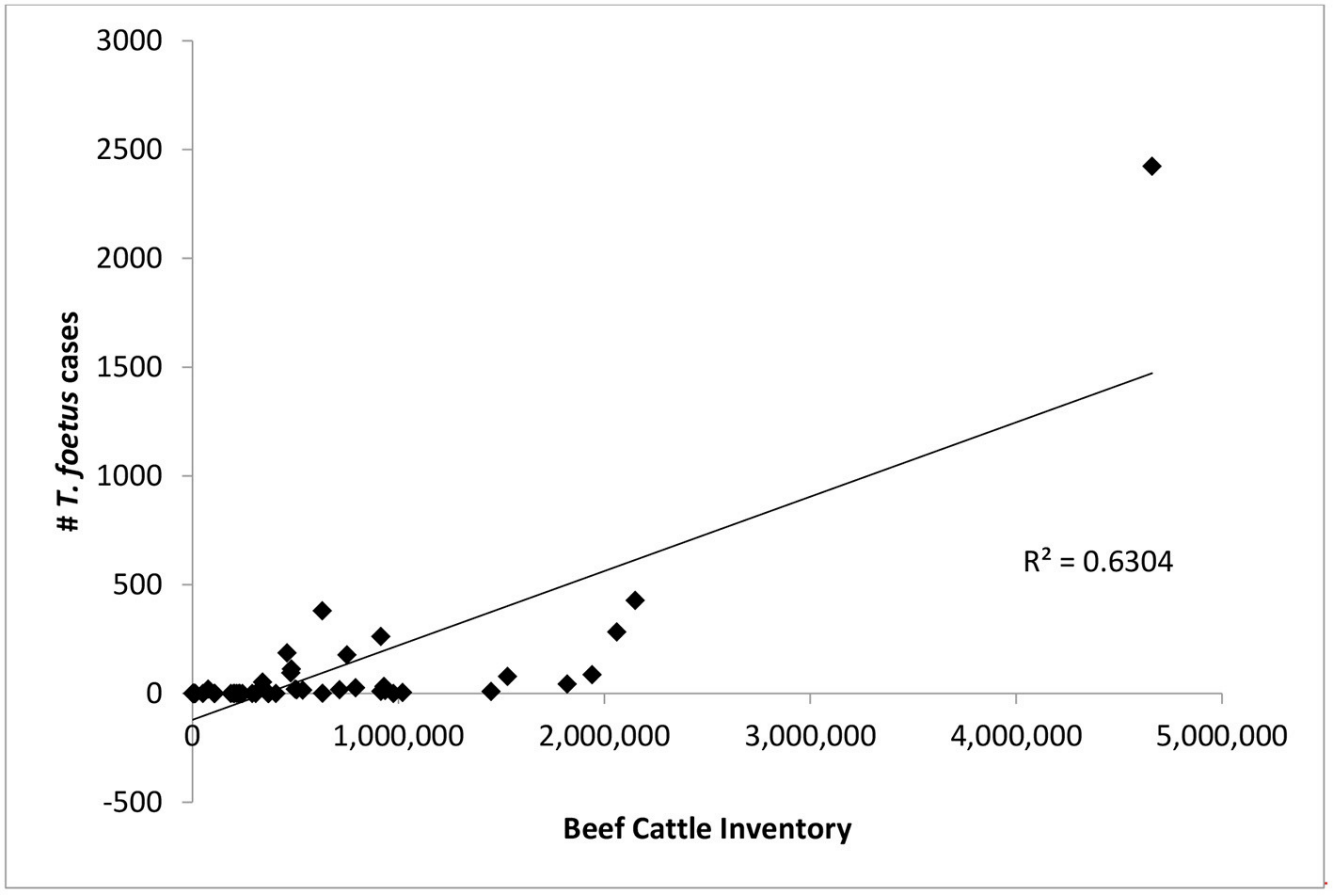
The number of bovine trichomonosis cases is compared to total number of beef cattle in each state. Points represent the total number of cases 2015–2019 for each state.

**Figure 4:**
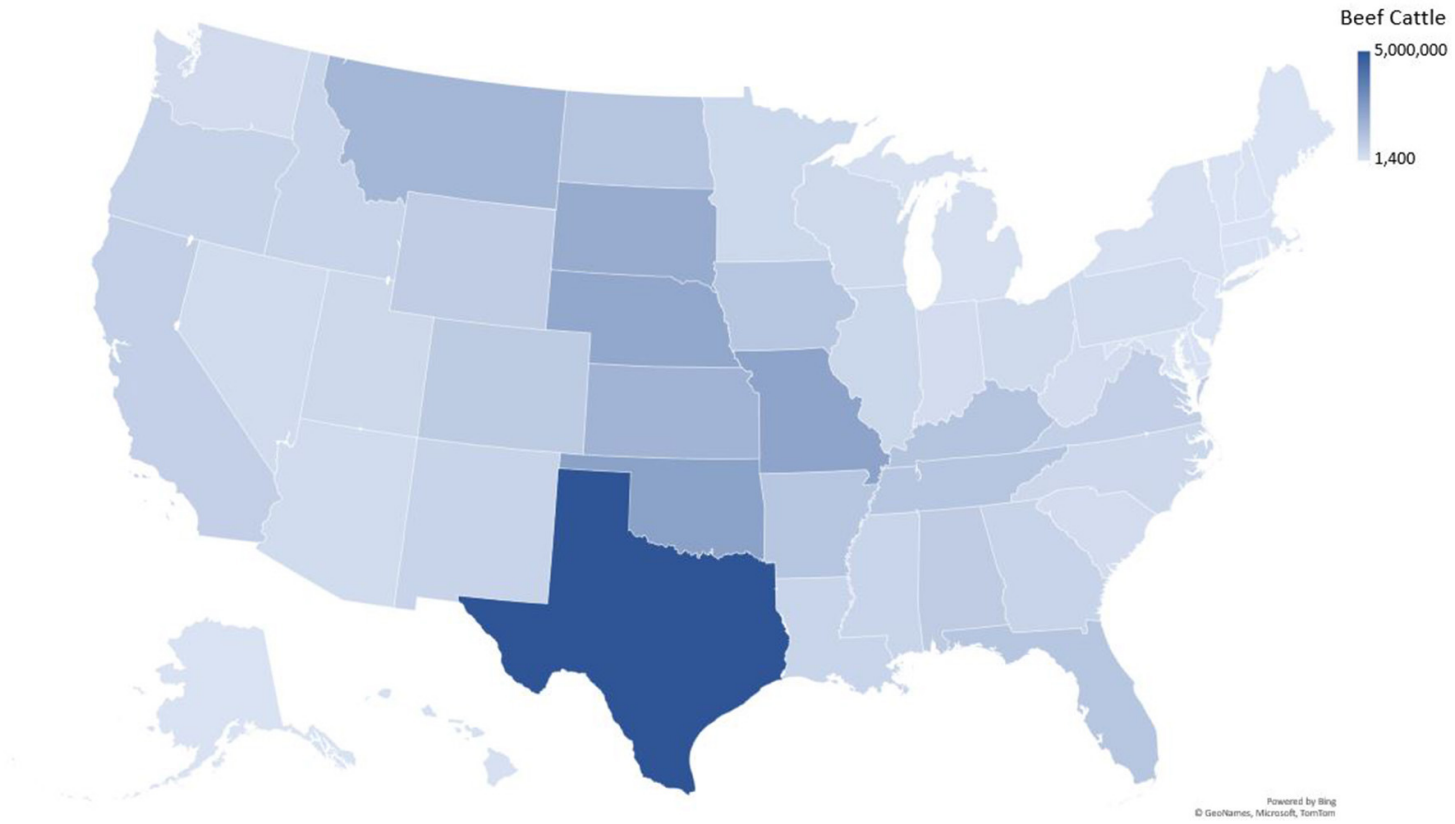
Beef cattle density in the United States.

There was only a weak correlation between total number of beef cattle in a state and the number of *T. foetus* cases reported (*R*^2^ = 0.63, Figure 3). However, this relationship was heavily influenced by Texas, which has both a large number of cattle and *T. foetus* cases. Beef cattle density and inventory are provided in Supplementary Table 1; Figure 4.

## Discussion

In cattle, *T. foetus* is a unique parasite since no persistent life stages occur in the environment. Therefore, a test and cull strategy has been proposed for eliminating the pathogen (7). Such a scenario is more feasible in states with a relatively low number of cases. States with a high number of cases may warrant a more comprehensive control program involving vaccination or additional surveillance. Prior to this study, there was not a comprehensive assessment of *T. foetus* case numbers throughout the United States. The purpose of our study was to assess the relative abundance and distribution of *T. foetus* in different states as the next step toward considering relevant control strategies.

Previous studies have attempted to capture the prevalence of *T. foetus* in individual states and regions of the United States. The methods of these studies vary, with some utilizing a retrospective approach and others testing a proportion of the state's bull population. Prevalence data from those studies is as follows: Idaho 11.25% (1996), Oklahoma 7.8% (1979), Colorado 7.5% (1964), Arizona 7.5% (1964), New Mexico 7.5% (1964), Utah 6.75% (1958, 1964), Florida 6.65% (1979, 1989, 1999, 2004), Nevada 6.1% (1964,1989), California 4.1% (1990), Wyoming 3.8% (1964, 2011), Texas 3.7% (2012), Alabama 0.27% (2008), Missouri 0.2% (1995), Nebraska 0.127% (1994), and Tennessee < 0.01% (2017) (7–22). The majority of these studies are significantly outdated and due to differences in methodology direct comparisons between states cannot be made in all cases.

There are two major challenges associated with determining the status of bovine trichomonosis in the United States: testing is significantly biased toward bulls and testing requirements vary significantly from state to state. Not only do these challenges impact the information our survey was able to gather, but also the conclusions that can be made based on the responses from state veterinarians. States where beef cattle production is limited are less likely to impose reporting or testing requirements for bovine trichomonosis. Because of the differences among states in the way testing is conducted and data is compiled, our study is not able to capture the actual incidence or prevalence of bovine trichomonosis. In general, diagnostic testing is focused on bulls and especially those that are being transported across state lines (23). Thus, testing is biased toward bulls and is often only carried out when required by law. Depending on state regulations, a positive test may or may not lead to testing of exposed animals. This means some states may have an increased number of positive cases due to follow-up testing.

Only 27 (0.7%) of the 3,817 positive test results included in this report are from cows, with 99.3% of all positive results being from bulls. A single positive bull could potentially infect many susceptible cows if it is not culled from the breeding herd. Since testing is biased toward bulls, the number of positive cases reported is likely significantly lower than the actual number of infected animals in the U.S. cattle population. The general recommendation for bull to cow ratios is 1 bull to 20–30 cows (24). A single mating between an infected bull and susceptible heifer has been shown to result in transmission 95% of the time (25). If every positive bull (3,790) in this report bred 20 susceptible females and 95% of those matings resulted in transmission of *T. foetus*, the total number of infections would be 72,010; this number still does not take into account bulls that are either not tested or positive tests in states where reporting is not required. The bias of testing toward bulls—particularly those being sold or moved across state lines, inconsistency of testing and reporting requirements, and the transient nature of infection in cows makes determination of prevalence or incidence in the cattle population of the United States difficult to accurately estimate. However, our data does allow the identification of states with virtually no *T. foetu*s (i.e., reportable status and zero reported cases from 2015 to 2019), those with sporadic cases, and those where the parasite is enzootic. Again, it should be noted that reporting is not required in all states. Therefore, states without reporting regulations (Table 1; Figure 2) could simply be unaware of positive cases. The data presented in this report is valuable as there are no comprehensive reports of the status of bovine trichomonosis in the United States.

It is not appropriate to compare absolute case numbers between states without accounting for differences in practices, regulations, and reportable disease status. Cattle inventories and husbandry practices vary from state to state and this can influence the risk of exposure to *T. foetus*. For example, 20 states reported zero cases, with reporting being required in 10 of these states. In general, states where cattle production, specifically beef cattle production, is not a significant industry are less likely to require testing or reporting of positive animals. Furthermore, the number of *T. foetus* cases was only weakly correlated with overall cattle inventory (Figure 3), suggesting that circumstances other than livestock density lead to the spread of the parasite. One possible explanation for the weak correlation is the differences in husbandry practices for beef cattle in different parts of the country, particularly when it comes to breeding practices. As long as best practices are followed, it is extremely rare for *T. foetus* to be transmitted through artificial insemination (AI) (5). This means that producers who more heavily utilize AI for breeding can effectively eliminate the risk of *T. foetus* for their herd. A previous study in Texas also reported a weak correlation between case numbers and cattle density and suggested factors such as comingled grazing and high bull to cow ratios as potential explanations (11, 15, 20). The age of the animal has also been reported to impact the likelihood of infection with *T. foetus*, with bulls over the age of 4 being more likely to carry the organism compared to younger bulls (26). It was not possible for our survey to capture the ages of positive animals and therefore conclusions about the impact of the age of bulls cannot be drawn from this data. Encouraging producers to remove older bulls from the breeding population could reduce the impact of trichomonosis, although this may not be an economically feasible option for many producers. The unique situation of each state should be considered for context when citing the results presented in this study.

The primary diagnostic methods for *T. foetus* are culture and PCR. The World Organisation for Animal Health (OIE) recognizes microscopy for morphologic identification, conventional PCR on clinical samples, conventional PCR in combination with culture, and real-time PCR as recommended and validated methods, depending on the specific purpose of the test (27). Culturing for *T. foetus* involves inoculating media with sample material (e.g., smegma or cervicovaginal mucus), incubating the culture at 30–37°C, and examining the culture over a period of several days for the presence of trophozoites. Commercial culture pouches are available in the United states and the OIE also provides the conditions for the commonly used culture media. For conventional PCR, the primer pairs TFR3 (5′-CGG-GTC-TTC-CTA-TAT-GAG-ACA-GAA-CC-3′)/TFR4 (5′-CCT-GCC-GTT-GGA-TCA-GTT-TCG-TTA-A-3′) and TFR1(5′-GTA-GGT-GAA-CCT-GCC-GTT-G-3′)/TFR2 (5′-ATG-CAA-CGT-TCT-TCA-TCG-TG-3′) can be used in combination for the best sensitivity and specificity (27–29). Minor groove binder probe real-time PCR based on the TFR3/TFR4 primer pair using non-cultured smegma or vaginal mucus samples has significantly higher sensitivity than conventional PCR and combination culture/PCR methods (27, 30). Commercially available real-time PCR kits containing TFF2 (5′-GCG-GCT-GGA-TTA-GCT-TTC-TTT-3′)/TFR2 (5′-GGC-GCG-CAA-TGT-GCA-T-3′) primers and TrichP2 (5′-6FAM-ACA-AGT-TCG-ATC-TTT-G-MGB-3′) probe have been extensively evaluated and found to be highly accurate (31). The OIE recommends the use of commercially available real-time PCR kits or the use of published protocols adapted to the laboratory running the test. Other tools that have not been thoroughly explored are point-of-care diagnostic assays that could be used for screening purposes in the field. Such assays have been used in research settings (32) but face challenges of sensitivity and specificity in the presence of various inhibitors present in smegma. Detecting *T. foetus* often has serious implications for the herd involved, so these challenges need to be overcome before point-of-care testing can receive more serious consideration. Rapid field testing by veterinarians could be a useful tool for mitigating economic losses where the parasite occurs more commonly and for better estimating disease prevalence.

The majority of *T. foetus* detection is currently achieved through PCR, while culture is used to a lesser extent. Both assays are typically conducted by state veterinary diagnostic labs. It is possible for veterinarians to culture trophozoites in-clinic; however, in-clinic results do not typically satisfy testing requirements for interstate movement. In addition, the number of states only accepting PCR as a valid diagnostic test is increasing. The American Association of Veterinary Laboratory Diagnosticians (AAVLD) is responsible for granting accreditation status to diagnostic laboratories in the United States. The AAVLD recommends that accredited laboratories follow the test validation standards outlined by the OIE (33). There are widely accepted sampling techniques, particularly for bulls (23, 34). Proper sampling techniques are important as the presence of urine has been shown to decrease PCR sensitivity (35). According to Code of Federal regulations, when testing is required for interstate movement, a Category II Accredited Veterinarian must be responsible for collecting and submitting samples to an approved diagnostic laboratory. When testing is required for reasons other than interstate movement, specific requirements are determined by the state of residence. Results from pooled sample submissions are considered acceptable by some states, while other states only accept individual sample results. Previous studies have shown that pooling samples at ratios up to 1 positive sample to 25 negative samples does not significantly reduce diagnostic sensitivity (36, 37). Requiring animals to be sampled by accredited veterinarians and have tests performed by accredited diagnostic labs helps to ensure consistency and confidence in test results.

While we could determine relative differences in disease status between states, the *T. foetus* cases reported in our survey only represent a proportion of cases in the country. Refinement of sampling and data collection techniques will improve the ability to determine the impact of bovine trichomonosis in the United States. Focusing resources on control of *T. foetus* in some affected regions could be a cost-effective investment toward eliminating economic losses associated with this parasite. Our study confirmed that bovine trichomonosis is still a significant pathogen in the US.

## Data Availability Statement

The original contributions presented in the study are included in the article/Supplementary Material, further inquiries can be directed to the corresponding author/s.

## Author Contributions

MB and KM conceived and designed the study and analyzed the data. JH collected the data. KM wrote the first draft of the manuscript. All authors read, revised, and approved the submitted version.

## Conflict of Interest

The authors declare that the research was conducted in the absence of any commercial or financial relationships that could be construed as a potential conflict of interest.

## Publisher's Note

All claims expressed in this article are solely those of the authors and do not necessarily represent those of their affiliated organizations, or those of the publisher, the editors and the reviewers. Any product that may be evaluated in this article, or claim that may be made by its manufacturer, is not guaranteed or endorsed by the publisher.
