# Supplementary material for: Bovine Trichomonosis Cases in the United States 2015–2019
Source: Front Vet Sci. 2021 Aug 9;8:692199. doi: 10.3389/fvets.2021.692199 (PMC8382153; doi:10.3389/fvets.2021.692199)
Supplement: Supplementary file 1 [file Table_1.docx]

| State | Beef cattle inventory |
| --- | --- |
| Alabama | 750,000 |
| Alaska | 6,800 |
| Arizona | 215,000 |
| Arkansas | 935,000 |
| California | 630,000 |
| Colorado | 792,000 |
| Connecticut | 4,500 |
| Delaware | 2,000 |
| Florida | 914,000 |
| Georgia | 499,000 |
| Hawaii | 75,500 |
| Idaho | 506,000 |
| Illinois | 405,000 |
| Indiana | 204,000 |
| Iowa | 930,000 |
| Kansas | 1,530,000 |
| Kentucky | 1,020,000 |
| Louisiana | 459,000 |
| Maine | 11,000 |
| Maryland | 50,000 |
| Massachusetts | 7,000 |
| Michigan | 108,000 |
| Minnesota | 370,000 |
| Mississippi | 477,000 |
| Missouri | 2,060,000 |
| Montana | 1,450,000 |
| Nebraska | 1,940,000 |
| Nevada | 242,000 |
| New Hampshire | 4,000 |
| New Jersey | 9,500 |
| New Mexico | 480,000 |
| New York | 105,000 |
| North Carolina | 367,000 |
| North Dakota | 975,000 |
| Ohio | 307,000 |
| Oklahoma | 2,150,000 |
| Oregon | 535,000 |
| Pennsylvania | 225,000 |
| Rhode Island | 1,400 |
| South Carolina | 186,000 |
| South Dakota | 1,820,000 |
| Tennessee | 914,000 |
| Texas | 4,660,000 |
| Utah | 340,000 |
| Vermont | 14,000 |
| Virginia | 631,000 |
| Washington | 230,000 |
| West Virginia | 198,000 |
| Wisconsin | 290,000 |
| Wyoming | 714,000 |

Table 1. Beef Cattle inventory by state.
