# Supplementary material for: Bovine Trichomonosis Cases in the United States 2015–2019
Source: Front Vet Sci. 2021 Aug 9;8:692199. doi: 10.3389/fvets.2021.692199 (PMC8382153; doi:10.3389/fvets.2021.692199)
Supplement: Supplementary file 2 [file Data_Sheet_1.docx]

State: __________________________

Name:__________________________

Position: ________________________

What is the estimated number of cattle within your state?

What is the average herd size in your state?

Is *Tritrichomonas foetus* reportable in your state?

What is the estimated prevalence of *T. foetus* in your state?

What are the estimated economic losses caused by *T. foetus* in your state?

What are the import regulations regarding *T. foetus* in your state? Is a negative T. foetus test required prior to import of breeding bulls and/or cows in your state?

How many positive T. foetus cases occurred in (total, number of bulls, number of cows):

2019?

2018?

2017?

2016?

2015?

What is the preferred method of testing in your state, PCR or culture?

Number of cases diagnosed by PCR:

2019:

2018:

2017:

2016:

2015:

Number of cases diagnosed by culture:

2019:

2018:

2017:

2016:

2015:

Are herds commonly co-mingled in your state? Do land permits for federal grassing overlap so that herds may comingle?

Is there a T. foetus “Control Program” in your state? Is vaccination a requirement?

Is there any additional information regarding *T. foetus* would you like to include?
